# Factors affecting general practitioners’ decisions to adopt new prescription drugs – cohort analyses using Australian longitudinal physician survey data

**DOI:** 10.1186/s12913-019-3889-4

**Published:** 2019-02-07

**Authors:** Yuting Zhang, Susan J. Méndez, Anthony Scott

**Affiliations:** Melbourne Institute: Applied Economic and Social Research, Faculty of Business and Economics, The University of Melbourne, 5th floor, FBE Building 111 Barry St, Carlton, VIC 3010 Australia

**Keywords:** Australia, Adoption of new prescription drugs, General practitioners

## Abstract

**Background:**

We investigate factors affecting Australian general practitioners’ decisions to adopt novel oral anticoagulants (NOACs) for the prevention of stroke/systemic embolism among patients with atrial fibrillation. Australia has a national homogeneous review and coverage system, which enables us to distinguish physician level factors while maintaining system level factors and patient coverage information constant.

**Methods:**

We conduct a cohort analyses by using longitudinal physician survey data from the Medicine in Australia: Balancing Employment and Life panel survey of Australian physicians (MABEL). MABEL data contain rich physician-level information such as age, gender, education, risk preferences, personality, physicians’ communications with other medical professionals, and other practice characteristics. Importantly, the survey data were linked, with physician’s consent, to actual utilization data from the Australian Pharmaceutical Benefits Scheme and the Medicare Benefits Schedule between January 1, 2012 and December 31, 2015. We measure speed (days until first time prescribing) of adopting NOACs. We estimate a Cox proportional hazard model to estimate factors affecting the adoption speed.

**Results:**

Several factors predict earlier adoption of NOACs: being male, more likely to take clinical risk, higher prescribing volume, being a principal or partner in the practice instead of an employee, spending less time in a typical consultation, and practicing in more affluent areas or areas with a higher proportion of older patients. GPs in Queensland are more likely to adopt NOACs and more likely to be extensive early adopters compared to other GPs. Other characteristics including physician personality, family circumstances, their involvement with public hospitals and teaching activities, and the distance between physician practice location to other clinics in the area are not statistically associated with earlier adoption.

**Conclusions:**

Our paper is one of the first to study the relationship between GPs’ risk preferences, personality and their decisions to adopt new prescription drugs. Because NOACs are commonly prescribed and considered more cost-effective than their older counterpart, understanding factors affecting physicians’ decisions to adopt NOACs has direct policy implications. Our results also highlight that even with universal coverage for prescription drugs, access to new drugs is different among patients, partially because who their doctors are and where they practice.

## Background

The successful adoption of new health technology that has been shown to be more cost-effective than the technology it replaces, is key to improving efficiency in health care. In an era of low value care and waste, the speed of adoption of cost-effective technologies can directly influence population health and healthcare costs. Even when new clear evidence exists and when costs are subsidised, there are many reasons why physicians continue to use old technologies, some of which are related to appropriate use in a small subset of the population, whilst other reasons are related to physician’s and patient’s beliefs and preferences and cognitive biases [1].

Prescribing accounts for a large proportion of healthcare expenditure in most countries, and some countries, including Australia, have strict decision rules on cost-effectiveness that determine the extent of public subsidy. For pharmaceuticals which have been shown to be cost-effective, understanding the extent to which these drugs are used in target populations has the potential to improve patient’s health at an acceptable cost, or maintain patient’s health at a lower cost, relative to existing old drugs used to treat the same condition. Conceptually, many factors both at the macro- and micro-level may affect the diffusion of a new prescription drug, including government agencies’ approval speed to make a new drug available in the market, the marketing efforts of pharmaceutical companies, physician demographic and professional characteristics, and patient characteristics [2, 3].

In this paper, we focus on how physician characteristics affect physicians’ decisions to adopt new prescription drugs. Specifically, we study novel oral anticoagulants (NOACs) for the prevention of stroke or systemic embolism among at-risk patients with non-valvular atrial fibrillation (NVAF), arguably one of the most innovative drug categories that affect many patients in recent years. Before the introduction of NOACs, for over 60 years warfarin was the only available oral anticoagulant to prevent stroke in atrial fibrillation. However, warfarin is difficult to take because it has a narrow therapeutic window and requires weekly blood monitoring and interacts with other drugs, some foods and concurrent illnesses. The introduction of NOACs has been considered as the beginning of a new era in anticoagulation in atrial fibrillation [4].

We use a dataset that includes rich data on physician characteristics from the Medicine in Australia: Balancing Employment and Life (MABEL) panel survey of Australian physicians. This unique dataset has been linked, with physician’s consent, to actual drug utilization data from the Australian Pharmaceutical Benefits Scheme (PBS) and the Medicare Benefits Schedule (MBS). Our paper contributes to the literature in several distinct ways. First, Australia has a national homogeneous review and coverage system, which enables us to distinguish physician level factors while maintaining system level factors and patient coverage information constant. Second, our data have rich physician-level information such as physician age, gender, education, risk preferences, personality, physicians’ communications with other medical professionals, and other practice characteristics. Some of these factors have not been studied before, because of the lack of available data. For example, attitudes to risk are considered as a fundamental part of decision making under uncertainty, and various types of physician behaviour are closely related to physicians’ risk attitudes. However, little is known about how physicians’ risk preferences affect their decisions to adopt new prescription drugs for their patients. This is partially because physicians’ risk preferences are difficult to collect and rarely linked with prescription data. Third, the general practitioner (GP) plays a central role in delivering health care in Australia and other countries and NOACs are commonly prescribed by GPs. Information about factors affecting Australian GPs’ decisions to adopt NOACs is useful for policy-makers to design appropriate strategies to guide the diffusion process to patients in need and promote more cost-effective prescribing habits.

## Background and settings

Medicare is Australia’s national universal tax-financed system of health insurance. In 2015 total health expenditure in Australia was just under 10% of Gross Domestic Product. Medicare provides subsidies to patients for out of hospital medical services provided by GPs and medical specialists; provides around half of the funding for public hospitals that are managed and funded by the eight Australian States and Territories; and subsidizes pharmaceuticals through PBS. The Federal government also subsidizes private health insurance premiums, but these do not cover GP visits or pharmaceuticals provided outside of hospitals. In Australia, the Australian Therapeutic Goods Administration (TGA) evaluates the safety and efficacy of new drugs before new drugs can enter the Australian market [5]. Once a drug is TGA registered, the Pharmaceutical Benefits Advisory Committee reviews a new drug to decide whether the drug should be subsidized by the PBS on the basis of the efficacy, cost-effectiveness, and safety of the new drug, relative to the existing drug on the PBS for the same indication [6]. After a drug is listed on the PBS, patients pay the same copayment, regardless whether a drug is new or old. Typically, there is a gap between a new drug first registered by the TGA to enter the Australian market and when it is actually listed on the PBS if approved for PBS coverage. Specifically, for the prevention of stroke and systemic embolism in patients with non-valvular atrial fibrillation, TGA approved dabigatran in April 2011, rivaroxaban in May 2012, and apixaban in April 2013. Rivaroxaban was added to the PBS on August 1, 2013, for the prevention of stroke in at-risk patients with NVAF on a cost-effectiveness basis in comparison with warfarin. On September 1, 2013, both apixaban and dabigatran were added to the PBS for the same indication on a cost-minimization basis to rivaroxaban with the equi-effective dose [7]. Once listed on the PBS, the use of NOACs has increased rapidly in Australia. For example, in 2015 alone, there were 1,604,242 PBS-subsidised prescriptions for NOACs supplied for 188,130 Australians with non-valvular atrial fibrillation [7].

Atrial fibrillation is the most common cardiac arrhythmia in adults and becomes more common with increased age, and is associated with a four to five times increased risk of stroke, and is directly responsible for 15–20% of all strokes and up to 36% of all strokes occurring in patients between 80 and 89 years old [8, 9]. Management of patients with non-valvular atrial fibrillation is aimed at reducing symptoms and preventing severe thromboembolic complications with adequate use of anticoagulant therapy such as warfarin or a NOAC.

Because oral anticoagulation agents prevent the risk of thromboembolic events, mainly strokes, through the thinning of the blood and delaying the coagulation process, they inherently increase the risk of bleeding, which may be minor (e.g., nose bleed) or life-threatening (e.g., intracerebral bleeding or gastrointestinal hemorrhage). For instance, the adjusted incidence of major bleeding could be as high as 9% among new NVAF US Medicare patients on dabigatran [10]. Oral or injectable vitamin K can be administered to reverse the effects of warfarin in major bleeding. However, during our study period, there were no antidotes available for NOACs in the event of major bleeding. In April 2016, the Australian Therapeutics Goods Administration approved an antidote to dabigatran, idarucizumab, for an implementation date of June 1, 2016. Currently no antidotes are available in Australia for other NOACs. Thus, the perceived risk of NOACs could be higher than warfarin, even though clinical trials have showed that NOACs generally do not have much higher bleeding risk than warfarin [11, 12].

Early research has shown that several physician characteristics are associated with early adoption of pharmaceuticals: physicians’ affiliation with a university/teaching hospital, larger practice size, and heavier category-level prescribing volume [13, 14]. A recent systematic literature review synthetized 35 studies and reported that some physician characteristics, in particular, fewer years in practice, larger prescribing volume – either in total or within the therapeutic class of the new drug, and more professional and social interactions among physicians are strong predictors for early adopters. In addition, physicians seeing younger patients, patients with higher socioeconomic status, and poorer health are more likely to prescribe new drugs early [15].

Although early adoption of some drug types may share some physician characteristics, it is not confirmed that early adoption of new drugs is a personal trait that is independent of drug type [16]. That is, even the strong predictors mentioned above do not consistently predict early adoption for all new drugs. Previous researchers find wide variations in initiating and choosing the type of anticoagulants, after the adjustment of patient characteristics [17]. This suggests a considerable amount of variation is due to physicians’ decisions in their judgment of perceived risks and benefits among their patients. However, little is known about physician’s adoption of NOACs – we only found one study that examined associations between a physician’s age, gender, medical school, specialty, and prescribing volume, and the adoption of dabigatran only before 2011 in Pennsylvania [18] and authors found cardiologists adopted dabigatran more rapidly than primary care doctors. To our knowledge, our study is the first national study to evaluate how Australian GPs adopt NOACs using rich physician information.

Moreover, our study is one of the first to evaluate how physicians’ risk preferences and personality affects their decisions to adopt new prescription drugs. Physician’s risk preferences are hypothesized to affect their prescribing behaviors. For example, Michel-Lepage and colleagues [19] used national French cross-sectional survey data to evaluate the relationship between GPs’ risk preferences and antibiotic prescribing and found that risk-averse GPs were more likely to follow guidelines and use more Rapid Antigen Diagnostic Tests in tonsillitis in children. Using similar data, Massin and colleagues [20] found that risk-averse GPs were more likely to be vaccinated against influenza themselves and to recommend influenza vaccination to their patients, compared to their more risk-tolerant peers. But we have not found any studies that examine how physician’s risk preferences and personality affect the speed at which physicians deicide to adopt new prescription drugs.

## Methods

### Data sources and study population

Our main data source is the Medicine in Australia: Balancing Employment and Life (MABEL) panel survey of medical practitioners. The first wave was in 2008 with 10 annual waves now completed or in progress. In total, around 10,000 medical practitioners (including approximately 3500 GPs) each year respond to a mailed invitation and doctors can complete a paper copy or log in to complete an online version of the survey. Respondents are broadly representative of the physician population in Australia [21]. Because most physician characteristics do not change over time, we use 2013 MABEL data to capture physician characteristics at the baseline when NOACs were first listed on PBS.

We have 1099 GPs in MABEL who signed consent forms in 2016 to link their PBS and MBS. We obtained all PBS and MBS items for these physicians between January 1, 2012 and December 31, 2015. We first selected our potential pool of GPs who would prescribe NOACs for NVAF by identifying those who prescribed warfarin before August 1, 2013, the date when the first NOACs was listed on PBS for patients with NVAF [*n* = 1034]. NOACs were listed on the PBS before August 1, 2013 for other indications than NVAF: the treatment of deep vein thrombosis, the prevention of venous thromboembolism, and for the prevention of recurrent venous thromboembolism. For this reason, we need to make sure prescriptions for NOACs were for NVAF only by using unique PBS item codes (2268J, 2691P, 2753X, 2769R, 2735Y, 2744 K). However, 2268 J for rivaroxaban 20 mg is also used for other indications besides NVAF, so we excluded 109 GPs who prescribed rivaroxaban for other conditions before August 1, 2013 [*n* = 925].

### Outcomes

Our main outcome is the speed of adopting NOACs. To measure the speed of adoption of NOACs, we defined the number of days until first time prescribing any of the three NOACs. Specifically, for each GP, we calculated the number of days from the PBS’s listing date to the first date when the GP prescribed a NOAC before December 31, 2015.

### Predictors

Informed by conceptual frameworks and empirical results from the prior studies, we include several physician personal and professional characteristics as predictors that may affect their decisions to adopt new drugs.

### Personal and medical training characteristics

Physician-level personal characteristics include gender, whether the physician lives with a partner, and whether she/he has children. Medical training includes: whether a doctor was trained in Australia or overseas, whether she/he was trained in one of the top eight medical schools in Australia, whether a doctor had a Fellowship of the Royal Australian College of General Practitioners or the Australian College of Rural and Remote Medicine, and the number of years practicing in medicine (1 < 10 years as reference group, 2 = 10 ≤ years< 20, 3 = 20 ≤ years< 30, 4 = 30 ≤ years< 40, and 5 = years≥40). Because the number of years in practice is highly correlated with age and prior studies more consistently show practice years predict adoption patterns, we include practice years instead of physician age in the model.

### Risk preference and personality

A particularly attractive feature of MABEL is that we can control for personality variables including risk preferences and the big-five personality traits. First, risk preferences are measured directly by multiple domains by adapting the Risk Propensity Scale proposed by Nicholson et al. [22] to the context of physician behaviours. We used the measure most relevant in adopting new drug - clinical risk. Specifically, physicians were asked directly about their everyday risk-taking on a five-point Likert scale where 1 is ‘very unlikely’ to 5 is ‘very likely’: “How likely are you to engage in clinical risks (e.g. recommending a treatment which is new to your usual practice or is controversial).” Because very few doctors answered 4 and 5, we merged doctors who answered 3 through 5 into one category. Second, personality traits of doctors were measured using the 15-item ‘Big Five’ factor model [23]. The five factors of personality traits include extraversion, agreeableness, conscientiousness, neuroticism and openness to experience.

### Physician practicing style

The physician practicing style is captured by prescribing volume, the length of a physician’s typical consultation measured in minutes, whether a physician worked any hours in public hospitals, involvement in teaching activities, and the standardized response to the degree to which the physician agrees (low values) or disagree (high values) to the statement: I normally consult with others in the practice about the management of patients with complex health and social problems. We defined prescribing volume as the average number of prescriptions (for all prescriptions as well as prescriptions in our category of interest) a physician wrote divided by the number of Level B consultations (typical type of GP consultants) she/he provided during our study period. The reason to divide the number of consultations is to adjust for the number and potential complexity of patients each physician saw. Because prescribing volume is highly skewed, we categorized it by quartile. Both prescribing volume variables on the basis of all prescriptions or category-level drugs are highly correlated and have a similar effect on adoption patterns, we therefore only include the prescribing volume for all prescriptions to capture whether a GP is a heavier prescriber in a broad sense.

### Social interactions and practice characteristics

The variables included in the physician practice characteristics are: whether the practice is accredited, the physician’s business role with the practice (reference group 1 = Principal or partner, 2 = associate, 3 = salaried or contracted employee, and 4 = locum or other), and three standardized index variables: patient complexity, practice network and communications with other professionals, and social isolation level. These three indexes were created using doctor survey questions in the MABEL. A patient complexity index was created on the basis of two questions: my patients have unrealistic expectations about how I can help them, and the majority of my patients have complex health and social problems. Practice network and communications index was created using answers from the question “I do not have a poor support of network of other doctors like me”. The social isolation index was created based on two questions: “I don’t have many friends or family members in my current work location”, and “It is easy to pursue my hobbies and leisure interests in my current work location.” All these questions were answered by a five-point scale: 1 = strongly disagree, 2-disagree, 3 neutral, 4 = agree, and 5 = strongly agree. We take the mean of the above variables used to create each index and then standardize them as mean zero and standard deviation one.

### Distance measures

For each GP in our sample, we calculated two distance variables using the location of all GPs and cardiologists in Australia: one measures the geodetic distance from the GP’s practice location to the nearest cardiologist (in kilometres) as a proxy for GP’s influence from cardiologists; the other measures the average kilometres from the GP’s practice location to the nearest three GP practice locations, which measures either the influence from other peer GPs, and/or the amount of competition among GPs.

### Area level characteristics

We controlled for different level of area characteristics available in the MABEL data. First, we adjusted deciles of two variables collected at the postcode level based on a physician’s work address: the index of relative socio-economic advantage and disadvantage (SEIFA), the proportion of population in the area older than 65. Second, we included the number of GPs per 1000 population at the Australian Statistical Local Area. Third, we included state dummies and dummies for the Remoteness Areas of the Australian Standard Geographical Classification (ASGC). These regional dummies control for unobserved factors that are constant within regions, such as the demography and characteristics of the population, the availability of health services and other providers, and marketing activity of pharmaceutical companies.

### Data analyses

We used a Cox proportional hazards model to evaluate simultaneously the effect of the above mentioned covariates on the rate of first-time prescribing at a particular point in time. We report the rate (commonly referred as the hazard ratio) associated with each covariate. In addition, we used a generalized linear regression (GLM) to estimate the association between physician characteristics and the number of days until first time prescribing among those who adopted during our study period. Cox and GLM Results are similar so we only report results from Cox model because the Cox model also account for censoring resulting from physicians who never prescribed any NOAC during our observational period.

This research has been approved by the University of Melbourne’s Human Research Ethics (Ethics ID: 0709559). Statistical software SAS 9.4 (Cary, North Carolina, USA) and STATA 15 (College Station, Texas, USA) were used for analyses.

## Results

### Generalization of our final study sample

**Fig. 1:**
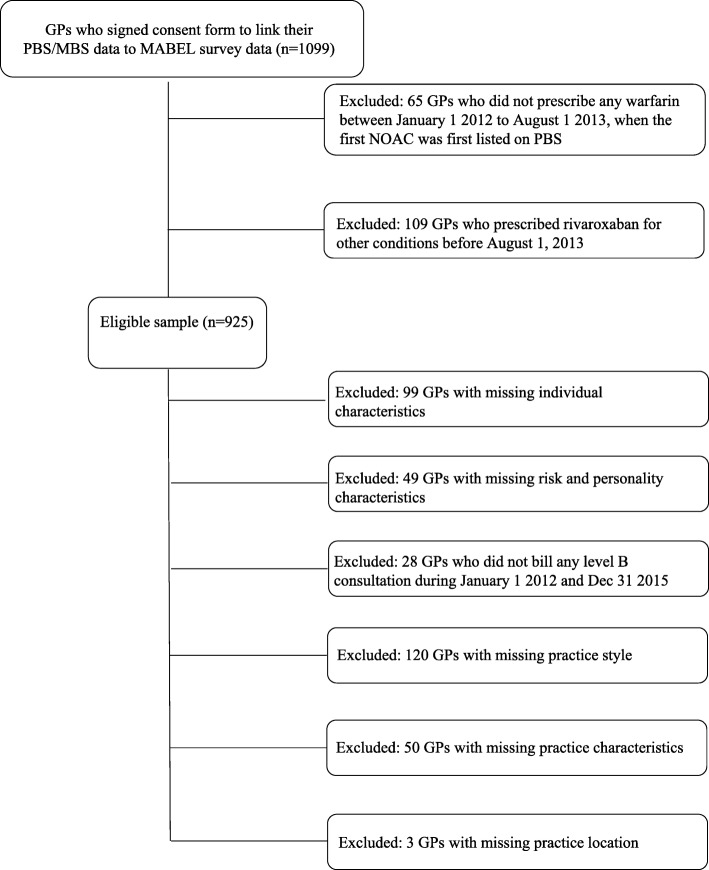
Flow diagram of study sample

Figure 1 shows the flow diagram on our sample selection. Our final sample includes 576 GPs who had no-missing values for all study variables. We compared our study sample with all Australian GPs in the Australasian Medical Publishing Company data and GPs who responded to MABEL (Table 1). Among the 25,000 GPs in Australia, 41% are female, their mean age is 54, and their distributions in each state are 33% in New South Wales (NSW), 25% in Victoria, 20% in Queensland, 10% in West Australia, and 8% in South Australia. Compared to the all Australian GPs, those who are female, younger, and those practicing in Victoria are more likely to sign consent forms to allow us to link their PBS/MBS data. Our final sample has similar state distribution as all MABEL GPs with consents.

**Table 1:** Generalizability of the Study Sample

	National GPs	MABEL GPs	Final Sample
	(*N* = 25,013)	(*N* = 1099)	(*N* = 576)
Male (%)	59	51	53
Age (mean)	54	51	53
State (%)			
New South Wales	33	26	27
Victoria	25	29	30
Queensland	20	18	17
Western Australia	10	10	9
Southern Australia	8	11	11

Notes: National GPs’ data are from the Australian Medical Publishing Company which only includes variables listed here. Base year: 2013. We have 1099 GPs who signed consent forms to allow us to link their MABEL survey data with actual prescription and medical data in the Australian Pharmaceutical Benefits Scheme and Medical Benefits Schedule

**Fig. 2:**
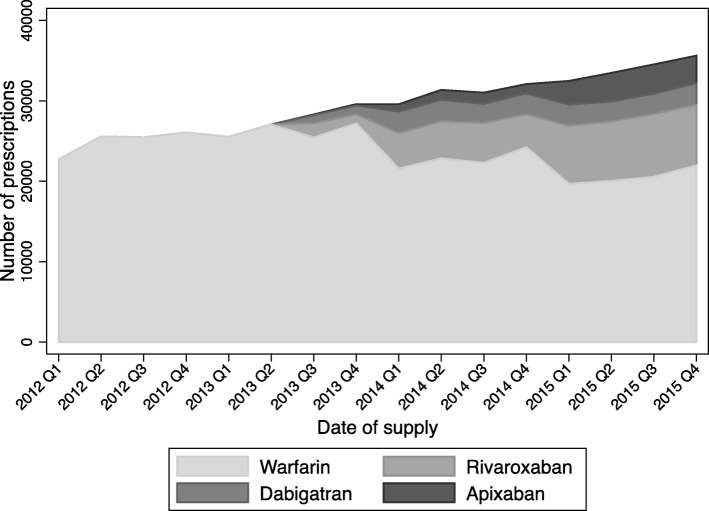
Number of anticoagulant prescriptions among MABEL general practitioners

Figure 2 shows the number of prescriptions written by GPs in our sample for each oral anticoagulant between January 1, 2012 and December 31, 2015. The prescribing pattern in our sample is similar as that among all Australian doctors shown in Fig. 1 in the recent national report [7]: over time, the use of warfarin has declined since PBS’s listing of NOACs for NVAF, but as of December 2015, warfarin remained the most used anticoagulant by number of prescriptions. Among three NOACs, Rivaroxaban is the most used, and dabigatran is the least used NOAC.

### Most physicians adopted a NOAC during our study period

**Fig. 3:**
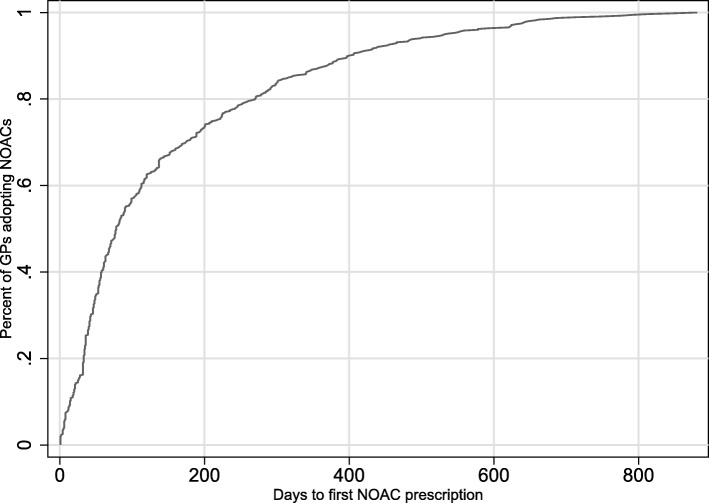
Cumulative distribution of days to first time prescribing a NOAC

Less than 4% of GPs (23 out of 576 GPs) did not adopt during our study period (28 months after PBS listing). Figure 3 shows the distribution of days to first prescribing among 553 adopters: the mean number of days to first time prescribing is 153 days, the median is 78 days, and 25th, 75th and 90th percentiles are 36, 216 and 400 days.

Table 2 summarizes characteristics of 553 GPs who adopted during our study period. About 53% of GPs are male, about half of GPs have longer than 30 years of practice experience, 90% of GPs live with partners, and 60% have children at home. About 19% of GPs were trained overseas, 20% of GPs were affiliated with public hospitals, and 63% of GPs were involved in teaching activities. On average our GPs spent 16 min in their typical consultation. Only about 4% of GPs said they were very likely to take clinical risk and 44% said they are very unlikely to take clinical risk. The mean distance to the closest cardiologist is 44 km and the average distance to the nearest three GPs is 4 km.

**Table 2:** Summary Statistics of General Practitioners Who Adopted NOACs During Our Study Period

Variable	Mean	sd
Main outcomes		
Days to first NOAC prescription	153	171
Individual characteristics		
Male	0.53	0.50
Years since graduation: less than 9 years	0.07	0.26
Years since graduation: 10–19 years	0.17	0.38
Years since graduation: 20–29 years	0.26	0.44
Years since graduation: 30–39 years	0.36	0.48
Years since graduation: more than 40 years	0.14	0.35
Overseas trained doctor	0.19	0.40
Top 8 Australian medical school	0.71	0.45
FRACGP or/and FACRRM fellowship	0.66	0.47
One or more children at home	0.60	0.49
Spouse	0.90	0.30
Risk and personality		
Clinical risk [1] very unlikely	0.44	0.50
Clinical risk [2]	0.36	0.48
Clinical risk [3]–[5] very likely	0.20	0.40
Openness	−0.15	0.95
Agreeableness	0.06	0.93
Conscientiousness	−0.05	1.02
Extraversion	−0.07	1.05
Neuroticism	−0.05	0.98
Prescribing volume		
Q1: Yearly average OAC prescriptions/Level B consultations	0.21	0.41
Q2: Yearly average OAC prescriptions/Level B consultations	0.25	0.43
Q3: Yearly average OAC prescriptions/Level B consultations	0.25	0.43
Q4: Yearly average OAC prescriptions/Level B consultations	0.29	0.45
GP practice style and practice characteristics		
Length of consultation (minutes)	16.30	4.64
Any involvement in teaching activities	0.63	0.48
Any involvement in public hospitals	0.20	0.40
Std. consult with others in practice	−0.05	1.01
Practice is accredited	0.96	0.20
Business relationship with practice: Principal	0.30	0.46
Business relationship with practice: Associate	0.11	0.31
Business relationship with practice: Salaried or contracted	0.56	0.50
Business relationship with practice: Locum or other	0.03	0.17
Std. practice support index	−0.02	1.02
Std. patient complexity index	0.01	1.01
Std. social isolation index	0.01	1.03
Distance to closest cardiologist in the state (Km)	44.22	144.90
Average distance to nearest 3 GP practices (Km)	3.98	11.09
Practice location characteristics		
SEIFA advantage and disadvantage (deciles at postcode level)	5.24	2.99
Pct. of population over 65 years old (deciles at postcode level)	6.12	2.85
No. of GPs per 1000 population (SLA level)	1.36	0.77
ASGC Remoteness Area: 1 Major city	0.63	0.48
ASGC Remoteness Area: 2 Inner regional	0.24	0.43
ASGC Remoteness Area: 3 to 5 Outer regional to very remote	0.13	0.34
State: New South Wales & Australian Capital Territory	0.29	0.45
State: Victoria & Tasmania	0.33	0.47
State: Queensland	0.17	0.37
State: South Australia	0.11	0.31
State: Western Australia & Northern Territory	0.10	0.30

Note: number of observation is 533

Abbreviations

NOAC = Novel oral anticoagulants

OAC = oral anticoagulants

FRACGP = Fellowship of the Royal Australian College of General Practitioners

FACRRM = the Australian College of Rural and Remote Medicine

SEIFA = Socio-Economic Indexes for Areas

SLA = the Australian Statistical Local Area

ASGC = the Australian Standard Geographical Classification

### Factors affecting the speed and intensity of adoption

Table 3 present results from the Cox model estimating factors affecting the speed of adoption measured by first-time prescribing. First, several physician characteristics strongly predict GPs’ decisions to adopt NOACs, including being male, higher prescribing volume, more likely to take clinical risk, and being a principal or partner in the practice instead of an employee. Specifically, male GPs are more likely to adopt NOACs than female GPs (hazard ratio = 1.51; 95% CI 1.22–1.86), after adjustment for other physician characteristics. GPs with modest tolerance to take clinical risk are more likely to adopt NOACs compared to GPs who self-reported to be very unlikely to take clinical risk, but the effect was not seen among GPs who reported very likely to take clinical risk. There is some evidence that the higher overall volume of prescribing is associated with quicker adoption, and the higher quartile of prescribing volume is associated with more likelihood of early adoption.

**Table 3:** Estimation Results From the Cox Model

	Days to first NOAC prescription	
	Hazard Ratio	[95% CI]
	(1)	(2)
Individual characteristics		
Male	1.511***	[1.224–1.864]
Years since graduation: less than 9 years (base)		
Years since graduation: 10–19 years	0.911	[0.604–1.375]
Years since graduation: 20–29 years	1.005	[0.674–1.500]
Years since graduation: 30–39 years	1.118	[0.754–1.658]
Years since graduation: more than 40 years	1.107	[0.703–1.741]
Overseas trained doctor	1.027	[0.715–1.476]
Top 8 Australian medical school	0.745*	[0.542–1.023]
FRACGP or/and FACRRM fellowship	1.001	[0.820–1.222]
One or more children at home	1.254*	[0.996–1.579]
Spouse	1.132	[0.827–1.548]
Risk and personality		
Clinical risk [1] very unlikely (base)		
Clinical risk [2]	1.236**	[1.008–1.514]
Clinical risk [3]–[5] very likely	1.214	[0.948–1.555]
Openness	0.910*	[0.818–1.013]
Agreeableness	1.020	[0.920–1.131]
Conscientiousness	0.933	[0.844–1.030]
Extraversion	0.984	[0.890–1.087]
Neuroticism	0.943	[0.855–1.041]
Prescribing volume		
Q1: Yearly average OAC prescriptions/Level B consultations		
Q2: Yearly average OAC prescriptions/Level B consultations	1.671***	[1.274–2.192]
Q3: Yearly average OAC prescriptions/Level B consultations	1.994***	[1.507–2.638]
Q4: Yearly average OAC prescriptions/Level B consultations	2.316***	[1.710–3.135]
GP practice style and practice characteristics		
Length of consultation (minutes)	0.953***	[0.931–0.975]
Any involvement in teaching activities	0.989	[0.798–1.224]
Any involvement in public hospitals	0.952	[0.732–1.238]
Consult with others in practice	1.039	[0.951–1.135]
Practice is accredited	1.495	[0.905–2.469]
Business relationship with practice: Principal (base)		
Associate	1.075	[0.778–1.486]
Salaried or contracted	0.658***	[0.519–0.835]
Locum or other	0.942	[0.541–1.641]
Std. practice support index	0.971	[0.887–1.063]
Std. patient complexity index	1.081	[0.984–1.188]
Std. isolation index	0.985	[0.902–1.076]
Distance to closest cardiologist in the state (Km)	0.999	[0.999–1.000]
Average distance to nearest 3 GP practices	0.999	[0.990–1.009]
Practice location characteristics		
SEIFA advantage and disadvantage (deciles at postcode level)	1.051**	[1.011–1.093]
Pct. of population over 65 years old (deciles at postcode level)	1.034*	[0.998–1.072]
No. of GPs per 1000 population (SLA level)	0.810***	[0.714–0.918]
ASGC Remoteness Area: 1 Major city (base)		
ASGC Remoteness Area: 2 Inner regional	0.888	[0.687–1.148]
ASGC Remoteness Area: 3 to 5 Outer regional to very remote	0.677**	[0.469–0.978]
State: New South Wales & Australian Capital Territory (base)		
State: Victoria & Tasmania	0.771**	[0.614–0.968]
State: Queensland	1.682***	[1.261–2.245]
State: South Australia	0.748*	[0.544–1.028]
State: Western Australia & Northern Territory	0.636***	[0.452–0.895]
Obs.	576	
Pseudo R-sqr		

Notes: Results are from a Cox proportional hazard model of days to first NOAC prescription. The number of observation is 576 including those who did not adopt a NOAC during our study period

***, ** and * indicate statistical significance at the one, five, and ten percent level

Abbreviations

NOAC = Novel oral anticoagulants

OAC = oral anticoagulants

FRACGP = Fellowship of the Royal Australian College of General Practitioners

FACRRM = the Australian College of Rural and Remote Medicine

SEIFA = Socio-Economic Indexes for Areas

SLA = the Australian Statistical Local Area

ASGC = the Australian Standard Geographical Classification

Second, GPs who spend more time in their typical consultation are slower in adopting NOACs: one extra minute spent in consultation is associated with 0.95 hazard ratio (95% CI 0.93–0.98) in the speed of adoption, and 0.92 odds ratio (95% CI 0.85–1.00) in the probability of being an extensive early adopter.

Third, areas where GPs practice affect their likelihood to adopt a NOAC. For example, GPs who practice in more affluent postcodes or areas with a higher proportion of older patients are more likely to adopt during our study period. GPs who work in areas with more GPs per 1000 population are less likely to adopt NOAC. GPs who work in remote regions are less likely to adopt NOAC, relative to those who work in major cities. There is also some state level variation after controlling for physician characteristics – relative to NSW, GPs work in Queensland are quicker to adopt a NOAC.

Fourth, other factors such as practice years, affiliated with public hospital and teaching activities, and social practice characteristics have no effects on adoption speed in our sample.

## Discussion

Physician adoption of new pharmaceuticals and innovation is a key issue for population health, especially where the new technology has been shown to be cost-effective. We find that males, a higher propensity to take clinical risk, heavy prescribing volume, being a principal or partner in the practice instead of an employee, spending less time in consultation, and practicing in affluent areas are GP characteristics predicting early adoption of NOACs. GPs in Queensland are more likely to adopt NOACs.

Our results on gender and prescribing volume are consistent with findings from previous studies for other drug classes. Physician prescribing characteristics are strong predictors for adoption patterns, whether we measure total number of all prescriptions, or prescribing volume in the therapeutic class of new drugs. However, for other factors that have been shown to be related to early adoption, such as affiliation with public hospitals, professional age, being involved in teaching activities, we did not find they affect the rate of adoption of NOACs among Australian GPs.

Our paper has several limitations. First, our ability to control for the effect of peer influences on prescribing is limited by using the distance from a GP’s practice location to the closest cardiologist and to other GPs. We have no direct measures of referral or peer networks [24]. Second, patient preferences and requests could affect doctors’ adoption patterns [25], but we only have limited information on patients. We include a measure of doctor’s perceptions of patient’s expectations. We also include a set of area characteristics to account for unobserved factors that drive demand for patient populations. Third, we do not have physician-level information on marketing efforts from pharmaceutical companies. This is partially controlled by area characteristics to the extent that market efforts vary by location. In addition, for the drugs we study, companies normally target cardiologists instead of GPs. A further note is that the drugs had been on the market for 2 years before they were first subsidized under the PBS, so the marketing efforts were no longer aggressive during our study period and may not differ much by GP. Similarly, we do not have data on the number of free samples distributed by each physician and on patient use of special access scheme and other compassionate use programs where medicines are made available before PBS subsidy starts. Fourth, we have a relative small sample size and our results may not be generalizable to physicians in other countries. In addition, there may be differences between Australian physicians and physicians in other places like US, Europe, Canada that may lead to subtle differences in which characteristics of prescribers predict uptake of new medications. This is first time researchers in Australia could link doctor survey data from MABEL with the actual pharmaceutical and medical data subsidized by Medicare, but only 1099 GPs among MABEL respondents signed consent forms to allow us to do this. We also wanted to include a comprehensive list of non-missing variables that could potentially affect NOAC adoption including the ones that have not been studied previously; unfortunately this further reduces our sample size. However, we tested the models with slightly larger sample sizes by including fewer covariates or treating missing values as a separate category - our key results are quite robust.

## Conclusions

In conclusion, we find several physician factors predict early adoption of NOACs: being male, a higher propensity to take clinical risk, heavy prescribing volume, being a principal or partner in the practice instead of an employee, spending less time in consultation, and practicing in affluent areas. Our study has some distinct contributions. First, this paper is one of the first to study the relationship between GPs’ risk preferences and their adoption decisions to new prescription drugs. Little is known about how physicians’ risk preferences affect their decisions to adopt new prescription drugs, because physicians’ risk preferences are difficult to collect and rarely linked with prescription data. Our study potentially fills this gap in the literature by showing modest effects of clinical risk attitudes on speed of adoption. Second, ours is one of the few studies that evaluate the effects of doctors’ personality on new drug adoption and we find personality does not affect adoption. Third, our findings suggest that involvement with public hospitals and teaching activities may not have large effects on the speed of adopting NOACs, after controlling for distances to cardiologists and other area-level variables. Finally, understanding factors affecting physicians’ decisions to adopt NOACs has direct policy implications since NOACs are considered more cost-effective than warfarin, and therefore the speed and intensity of adoption will have a stronger relationship to health outcomes. However, this is not as relevant in countries where NOACs cost a lot more than warfarin and therefore may not be considered cost-effective relative to warfarin especially among patients who are well controlled on warfarin. Our results also highlight that even though in a country with universal coverage for prescription drugs, access to new drugs is different among patients, partially because who their doctors are and where they practice.

## Abbreviations

ASGC: The Australian Standard Geographical Classification
FACRRM: The Australian College of Rural and Remote Medicine
FRACGP: Fellowship of the Royal Australian College of General Practitioners
GLM: Generalized linear regression
GP: General practitioner
MABEL: The Medicine in Australia: Balancing Employment and Life panel survey
MBS: Medicare Benefits Schedule
NOAC: Novel oral anticoagulants
NVAF: Non-valvular atrial fibrillation
OAC: Oral anticoagulants
PBS: Australian Pharmaceutical Benefits Scheme
SEIFA: Socio-Economic Indexes for Areas
SLA: The Australian Statistical Local Area
TGA: The Australian Therapeutic Goods Administration
